# Supplementary material for: Venomix: a simple bioinformatic pipeline for identifying and characterizing toxin gene candidates from transcriptomic data
Source: PeerJ. 2018 Jul 31;6:e5361. doi: 10.7717/peerj.5361 (PMC6074769; doi:10.7717/peerj.5361)
Supplement: Supplemental Information 6 [file peerj-06-5361-s006.gz › FinalOutput_1E-6/Venom_allergen_3_30/finaltree.pdf]

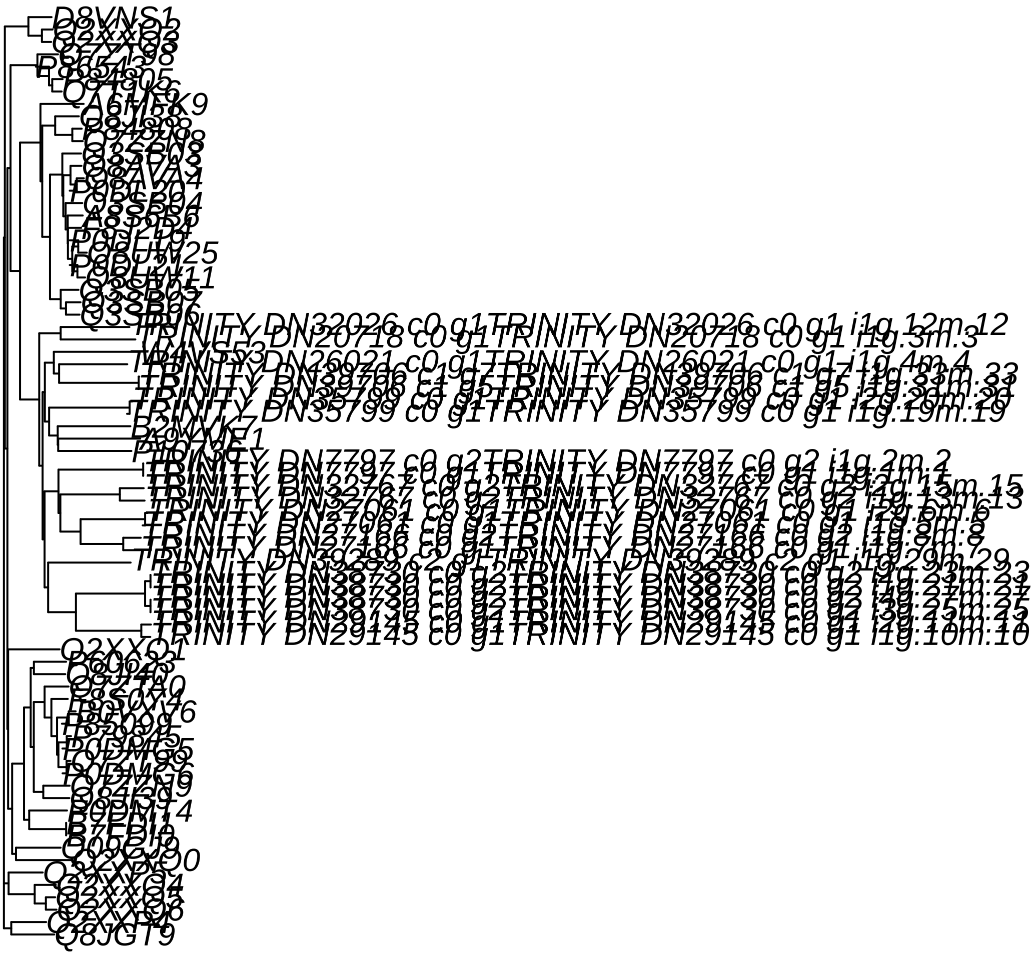
D8VNS1
A6MFK9
TRINITY DN32026 c0 g1TRINITY DN32026 c0 g1 i1g.12m.12
TRINITY DN20718 c0 g1TRINITY DN20718 c0 g1 i1g.3m.3
TRINITY DN26021 c0 g1TRINITY DN26021 c0 g1 i1g.4m.4
TRINITY DN35799 c0 g1TRINITY DN35799 c0 g1 i1g.19m.19
B2WVK7
TRINITY DN7797 c0 g2TRINITY DN7797 c0 g2 i1g.2m.2
TRINITY DN29289 c2 g1TRINITY DN29289 c2 g1 i1g.29m.29
TRINITY DN29145 c0 g1TRINITY DN29145 c0 g1 i1g.10m.10
Q2XXQ1
Q2XXQ0
Q8JGT9
